# Understanding public engagement in animal welfare in South Korea: a theory of planned behavior approach

**DOI:** 10.3389/fvets.2025.1657203

**Published:** 2025-11-17

**Authors:** Seola Joo, Myung-Sun Chun, Hyomin Park

**Affiliations:** 1 Research Institute for Veterinary Science, College of Veterinary Medicine, Seoul National University, Seoul, Republic of Korea; 2 Department of Urban Sociology, College of Urban Science, University of Seoul, Seoul, Republic of Korea

**Keywords:** animal welfare, policy, theory of planned behavior, animal attitude scale, structural equation modeling, self-efficacy

## Abstract

**Introduction:**

Human-animal relationships have changed significantly in recent decades, becoming increasingly diverse and ethically complex, thereby prompting increased societal concern for animal welfare. This study investigates public perceptions of animal welfare levels and related policies in South Korea, as well as the psychological and contextual determinants of pro-animal behavior as animal welfare engagement, employing the Theory of Planned Behavior (TPB) as its theoretical framework.

**Methods:**

A nationally representative online survey was conducted with 2,000 South Korean adults. Measures included attitudes toward animals, subjective norms, internal and external efficacy, behavioral intentions, and self-reported pro-animal behaviors. Structural equation modeling (SEM) was employed to test hypothesized relationships among TPB constructs and behavioral outcomes.

**Results:**

The findings indicate strong public demand for appropriate and effective political action on animal welfare issues. SEM results show that both pro-animal attitudes and internal efficacy significantly predict behavioral intentions, whereas subjective norms and external efficacy do not exhibit significant effects. Internal efficacy demonstrates both direct and indirect positive influences on pro-animal behavior. In contrast, external efficacy shows no statistically significant direct impact.

**Discussion:**

Public concern for animal welfare in South Korea is increasing, and internal efficacy and pro-animal attitudes play crucial roles in promoting behavioral engagement in animal welfare. Although external efficacy and subjective norms show limited influence, this does not imply that legislative efforts lack value. Rather, institutional support may enhance pro-animal behavior indirectly by strengthening individual confidence, underlining a potential mediating role of internal efficacy between external efficacy and behavioral outcomes. Findings emphasize the need for policies and educational initiatives that enhance individual confidence and motivation while complementing broader institutional frameworks. Future research should incorporate policy feedback theory to better understand the interaction between institutional context and public behavior.

## 1 Introduction

Animals have become an integral part of human society over recent decades, resulting in increasingly diverse and complex human-animal relationships. Human-animal interactions extend beyond conventional relationships with livestock and wildlife to encompass more intimate associations with companion animals and those occupying liminal spaces between domestication and wildness. There has been a marked shift in people’s behavioral choices regarding animals, as evidenced by the significant increase in the percentage of the population adopting a vegan diet over the past few decades (1, 2) and the growth of total expenditure in the companion animal industry from $90 billion in 2018 to an estimated $150 billion in 2024 (3).

Meanwhile, debates persist regarding the desirable form of human-animal relationship and standards of animal welfare. Public attitudes diverge widely on animal-related issues such as animal testing, factory farming, recreational hunting, and dietary choices including veganism. Even self-identified animal welfare advocates show varying levels of commitment to pro-animal behaviors, ranging from strong to minimal engagement (4).

In these dynamics, the South Korean government has established and implemented a Comprehensive Animal Welfare Plan every 5 years to enhance the protection and management of animals. The ongoing Third Plan, following the First and Second National Animal Welfare Plan, marks a paradigm shift from a passive model of “protection” to a proactive framework of “welfare.” Key strategies of the plan include reinforcing the implementation of existing policies, introducing preventive measures against abuse and neglect, strengthening collaboration with civil society organizations, and cultivating broader public consensus on the importance of animal welfare (5).

Park and colleagues (6) argue that animal welfare policy in Korea has largely been driven by economic efficiency and the instrumental values of industrial capitalism, often through government-led benchmarking of other developed countries or responses to specific issues (e.g., dog meat, animal farm certification). This contrasts with policy approaches that are grounded in social values concerning animals and fostered by close communication and broad public support (6).

Moreover, there is a lack of studies on the overall perception, purpose, and values of animal welfare policy as perceived by the public and various animal welfare related experience in Korea, although several studies have examined public understanding of animal welfare policies globally (4, 7–10). Existing Korean research has primarily focused on tracking trends and providing recommendations for animal welfare policies (6, 11), or on public surveys regarding specific animal welfare issues or programs, such as laying hen welfare or the purchase of animal welfare–certified products (12–14). A recent study (15) analyzed key findings from the 2023 Public Awareness Survey on Animal Welfare; however, the analysis was largely descriptive and lacked a theoretical framework to interpret the survey results.

For successful and effective implementation, a policy should be accompanied by public behavior. Policy refers to a set of strategies or directives determined and implemented by a government to address social issues (16). Accordingly, a country’s animal policy like Korean Comprehensive Animal Welfare Plan can be understood as the government’s strategies and directives aimed at resolving issues related to animals. The field of animal welfare policy emerged in the socio-political domain, in which active public participation serves as a driving force for policy reform (17). Animal welfare policies are increasingly shaped by social perceptions of animals and their socio-economic value, and policymakers are tasked with defining public goals, advancing collective interests, and crafting behavioral guidelines that align with these evolving perceptions.

A policy consists of three key elements: policy goal, target group, and policy instrument. Policy goal is a product of value judgment and serves as a guiding reference point for achieving social consensus. The target group refers to those affected by and involved in the policy implementation process. In the context of animal issues, the general public plays a key stakeholder role through consumption, opinion formation, and political engagement. Policy instruments are the practical means employed to achieve policy goals, requiring causal knowledge about the issue at hand (16).

Bryant et al. (8) examined the animal welfare legislation of 23 countries and found a strong correlation between public support for animal welfare and the enforcement of stricter farm animal welfare regulations, suggesting a reciprocal relationship between public attitude and institutional interventions. In other words, public opinion can drive the enactment of stronger animal welfare regulations, and these regulations, in turn, can shape public attitudes. While institutional-level interventions are generally considered more effective than individual-level actions due to their broader impact (18), when institutional efforts are complemented by active public participation, they are likely to achieve greater success in advancing animal welfare policies (8). Accordingly, the development and implementation of animal-related policies are often underpinned by collaborative governance among government agencies, local authorities, and private-sector actors such as animal advocate groups (17). In this context, insights into public perceptions of animal welfare and the socio-psychological motivations and barriers influencing public engagement with animal welfare initiatives are essential for the effective and sustainable development of policies aimed at enhancing animal welfare (19, 20).

Since complex and often conflicting human-animal relationships intersect in current society, simplified or top-down policy responses are insufficient for effective problem-solving. Nonetheless, animal policy remains largely institution- and expert-driven, with limited empirical understanding of public perceptions and behavioral tendencies. As Chen (4) emphasizes, aligning public opinion with policy objectives and design is crucial to policy success. Understanding public attitudinal and behavioral orientations toward animals and their welfare provides an important context for interpreting behavior and decision-making processes for animal welfare.

Explaining human behavior, however, is not a simple task; indeed, the field of behavioral sciences is dedicated to elucidating how people’s beliefs and attitudes toward social objects translate into observable actions. Some theoretical frameworks contend that human behaviors are fundamentally rooted in personality or disposition traits (21, 22), while others emphasize human behaviors as products of the actor’s normative context (23), wherein individuals strategically maximize utility within given situations (24). More recent approaches posit that rational calculation is not the sole driver of behavior; rather, diverse social factors regulate the path from an individual’s motivation to behavior. The incorporation of these varied social factors into models linking initial attitudes to behavioral outcomes has substantially enhanced predictive accuracy in understanding human behavior.

One such model is the theory of planned behavior (TPB), which explains how individual beliefs, specifically attitudes, perception of social norms, and perceived control over social contexts, lead to behavioral outcomes (25, 26). This study applies the TPB framework to examine whether people’s behaviors toward animals result from reasoned decisions shaped by both individual perceptions and social context. Specifically, this study hypothesizes causal relations among individual beliefs, the intention to consider animal welfare, and actual behavioral outcomes based on TPB, and employs structural equation modeling (SEM) on data from a national survey conducted in South Korea to empirically verify the hypotheses. Based on analysis results, this study elucidates the key factors driving pro-animal behavior and suggests nudging points for behavioral change that can inform future animal welfare policies.

To our knowledge, this study is the first to empirically investigate public perceptions of animal welfare policy in Korea and to analyze the psychological mechanisms linking individuals’ attitudes and perceptions to the formation of behavioral intentions and engagement with animal welfare. By doing so, the study aims to build a comprehensive and evidence-based foundation and to support the development of more effective policy instruments for advancing animal welfare. Although a substantial body of research examined people’s attitudes toward animal welfare, few studies have explored this topic through the TPB. This approach provides us with insight into the social psychological mechanisms underlying pro-social behavior. Moreover, it allows for an examination of how individuals’ perceptions of macro-level structures influence micro-level behaviors.

## 2 Theoretical background

### 2.1 Theoretical gap between attitudes and behavior in animal welfare

Public surveys support the refinement of animal-related polices by assessing attitudes, experiences, and perceptions related to animal welfare and/or animal welfare policies. Examples include the biannual Eurobarometer on Animal Welfare survey and the Animal Tracker Survey in the United States and Australia. These surveys measure public knowledge of animal welfare, levels of advocacy for animal protection, attitudes toward education and legislative goals, consumption of animal products, and beliefs about animal cognition and emotions (7). In South Korea, the Ministry of Agriculture, Food, and Rural Affairs conducts an annual survey on animal welfare to inform policy development (27). This survey addresses issues such as pet ownership rates, knowledge of animal welfare laws, attitudes toward animal abuse, pet adoption practices, perceptions of abandoned animals and shelters, and awareness of laboratory and farm animal welfare.

However, despite the plentiful data from animal welfare related surveys, many studies examining public awareness and behavior regarding animal welfare lack a clear theoretical framework, and few provide deeper insights that can help predict animal welfare-related behaviors, understand motivational barriers, and address the gap between behavioral intentions and outcomes.

Notwithstanding increasing concerns and interests in animal welfare, a discrepancy remains between expressed attitude and actual behavior (28, 29). This “attitude-behavior gap” has reported in across domains such as ethical consumption and pro-environmental behavior (30–32). Furthermore, the role of policy initiatives in shaping public attitudes or behaviors remains underexplored, limiting our ability to assess the socio-political impacts of animal welfare policies (9, 33).

Most importantly, research has yet to provide a comprehensive understanding of the causal relationship between people’s beliefs in animal welfare and their behavioral outcomes—knowledge essential for developing institutional measures aimed at enhancing public awareness and promoting pro-animal behaviors.

The present study addresses these gaps by investigating what determines individuals’ active engagement in pro-animal behavior. Specifically, this study employs TPB (25, 34), a well-established socio-psychological model for identifying psychological factors that influence individuals’ decisions to engage in related behavior via intention.

### 2.2 Theory of planned behavior (TPB)

According to the theory of reasoned action (TRA), an individual’s intention is determined by two core constructs: attitude (the person’s positive or negative evaluation of performing the behavior) and subjective norm (the perceived social pressure to perform or not perform the behavior) (35, 36). A favorable attitude toward the behavior and a stronger subjective norm (i.e., feeling it important that others think one should do it) lead to a stronger intention to perform the behavior. However, TRA does not account for the possibility that even with strong intentions, a person may be unable to carry out the behavior due to external constraints or lack of control. This limitation motivated an extension of the theory to better predict behaviors in the face of such constraints.

TPB extends the TRA model to offer a more comprehensive understanding of human behavior by identifying key behavioral determinants and barriers (25, 34, 37). The model gained significant attention in the field of behavioral sciences, since it bridged the gap between attitudes and behavior by incorporating additional factors influencing intention. The major extension of TPB from TRA is the introduction of perceived behavioral control (PBC) as another antecedent of intention (25, 38).

**Figure 1:**
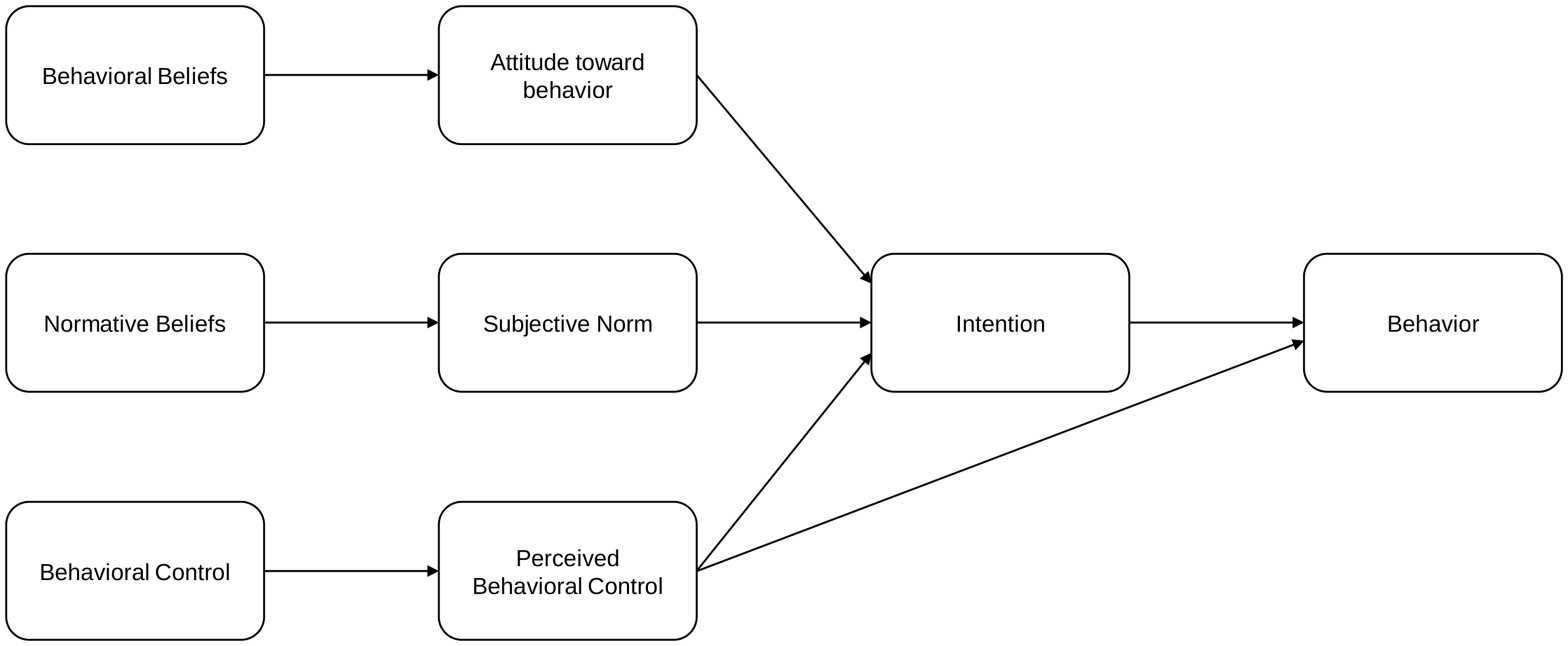
Theory of planned behavior (TPB) framework.

In the TPB model, PBC refers to the perceived ease or difficulty of performing a given behavior, reflecting the extent to which individuals believe that the behavior is within their control. This means that when a person tries to behave following their beliefs, opportunities and resources play a pivotal role in enhancing or reducing the likelihood of behavioral achievement. Therefore, by adding PBC to the model, TPB explains that human behavior is a mixture of behavioral intention and the ability to control the opportunities and resources. This helps explain why individuals with strong intentions may fail to act in accordance with what they believe is the right thing to do if they feel a lack of control. Previous research employing TPB has used measures of perceived self-efficacy, defined as the “judgement of how well one can execute courses of action required to deal with prospective situation” (39), to assess PBC. Moreover, in research on political behavior, some studies have applied measures of political efficacy within the PBC domain of the TPB model (40–42). Specifically, Alscher and Jana (40) distinguish between internal and external political efficacy, using trust in government or related institutions as an indicator of external efficacy. Importantly, they argue that efficacy should not be seen simply as a stable personal trait. Rather, people’s sense of influence is shaped by how actively governments or organizations respond to their concerns. Thus, external political efficacy is conceptualized not as a fixed, internalized disposition but as something shaped by external factors such as politicians’ actions or the design of institutional structures (40–43). This extension significantly enhances the model’s applicability to complex behaviors by accounting for constraints on action. Figure 1 illustrates the overall structure of the TPB model.

Another advantage of the TPB model is its ability to address multiple components of behavioral decision-making—attitude, subjective norm, control, and intention—each of which can serve as a potential intervention point for influencing behavior. The relative influence of these components may vary. If either attitude or social norm strongly favors a behavior, the effect of perceived control on intention may become relatively weaker, and vice versa (44). In other words, these factors can compensate for one another to some degree. By encompassing all three predictors, TPB provides a parsimonious and effective explanation of human behavior.

The TPB model’s effectiveness has been consistently validated through empirical research, confirming its capacity to account for a significant portion of variance in actual behaviors across diverse contexts. The model presents a versatile and robust framework for predicting regulated behavior, grounded in explicit assumptions about how intentions form and translate into actions to render its propositions testable and its structure parsimonious. Moreover, while TPB focuses on a few key antecedents of behavior, its flexibility enables the incorporation of additional variables relevant to a specific context when necessary (45). For instance, researchers might include moral values, past behavior, or habit as extra predictors in a TPB model for certain contexts. This ability to extend the model without altering its core structure allows TPB to maintain high predictive power across a wide range of behavioral domains. Due to this parsimony and strong explanatory power, the TPB has been successfully applied in many domains of human activity, from consumer decision-making, voting behavior, health behavior, environmental conservation actions to public transportation usage and food choices—demonstrating high accountability in explaining outcomes (33, 44). Such research provides valuable insights for policymakers, supporting the refinement of existing policies and development of new policies and programs aimed at encouraging behavioral change and improving outcomes (46).

Relevant to this research, TPB has also been applied to examine animal welfare-related decisions and behaviors. For instance, TPB has been used effectively to understand pro-animal behaviors such as purchasing animal welfare products (33, 47, 48) and farmers’ consideration and adoption of sustainable practices for animals (49, 50). In the domain of animal welfare, people’s attitudes toward animal welfare can vary by a multitude of factors. These factors include demographic characteristics (e.g., gender, age, income), group affiliation (e.g., farmers vs. pet owners vs. laboratory researchers), cultural and media influences, available resources, personal dietary habits, and even the specific animal species being considered (4, 9, 51).

TPB can accommodate these differences by examining how attitudes, subjective norms, and perceived control are shaped by such factors. Furthermore, it can provide insights into why individuals support or oppose animal welfare initiatives, allowing researchers to gain a better understanding of causal relations that lead to particular behaviors towards animals and design more effective interventions to promote animal welfare-friendly practices.

### 2.3 Structural pathways (hypothesis)

Drawing upon the TPB framework, we formulated hypotheses that articulate causal relations among individual beliefs, behavioral intention, and behavioral outcome regarding animal welfare. First, we posited that TPB’s three antecedents—behavioral beliefs, normative beliefs, and behavioral control—are reflected in an individual’s attitude toward animals, subjective norm regarding animal welfare, and perceived efficacy regarding animal welfare, which thereby impact an individual’s intention to consider animal welfare. Next, we hypothesized a direct causal relationship from intention to behavior, proposing that stronger intentions to consider animal welfare leads to increased engagement in pro-animal behaviors. Lastly, consistent with TPB, we hypothesized that behavioral control exerts both a direct effect on behavior itself, as well as an indirect effect through intention.

The specific hypotheses set based on TPB are as follows. The first two hypotheses (H1 and H2) predict the positive effects of individual beliefs, represented by attitude and subjective norm, on the intention to consider animal welfare. In South Korea, which is known for its strong collectivist culture, perceived subjective norms are expected to exert a significant influence on behavioral outcomes (52).

*H1. Attitude toward animals has a positive effect on the intention to consider animal welfare.*

*H2. Subjective norm regarding animal welfare has a positive effect on the intention to consider animal welfare.*

The next set of hypotheses reflects the relationship linking behavioral control to behavioral intention and outcome. Previous studies employing TPB use measures of self-efficacy for assessing PBC (44, 49), while studies on political efficacy distinguishes the concept of efficacy into internal and external efficacies (53, 54). In the context of political behavior, internal efficacy refers to an individual’s belief in their own ability to participate effectively in political engagement, and external efficacy refers to the belief that the political system is responsive to individual and collective influence (53). Applying these distinctions to the domain of animal welfare, we can conceptualize internal efficacy as an individual’s belief in their capacity to influence animal welfare effectively and external efficacy as the belief that the systems related to animal welfare are responsive to individual and collective influence. By distinguishing between internal and external efficacy, we aim to provide a more refined analysis of whether perceptions related to personal agency or institutional responsiveness are more salient in the context of pro-animal behavior, as well as how these two dimensions of efficacy may be interrelated. Based on these theoretical foundations, we set the following hypotheses:

*H3. Perceived efficacy regarding animal welfare has a positive effect on the intention to consider animal welfare.*

*H3-1. Internal efficacy has a positive effect on the intention to consider animal welfare.*

*H3-2. External efficacy has a positive effect on the intention to consider animal welfare.*

According to TPB, behavioral outcome is determined by both behavioral intention and PBC, the latter often operationalized through measures of efficacy. Within the TPB framework, behavioral intention is understood to mediate the relationship between its antecedents and the resulting behavior. Accordingly, we proposed the additional two hypotheses (H4 and H5) as follows.

*H4. The intention to consider animal welfare has a positive effect on pro-animal behavior.*

*H5. Perceived efficacy on animal welfare has a positive effect on pro-animal behavior.*

*H5-1. Internal efficacy has a positive effect on pro-animal behavior.*

*H5-2. External efficacy has a positive effect on pro-animal behavior.*

**Figure 2:**
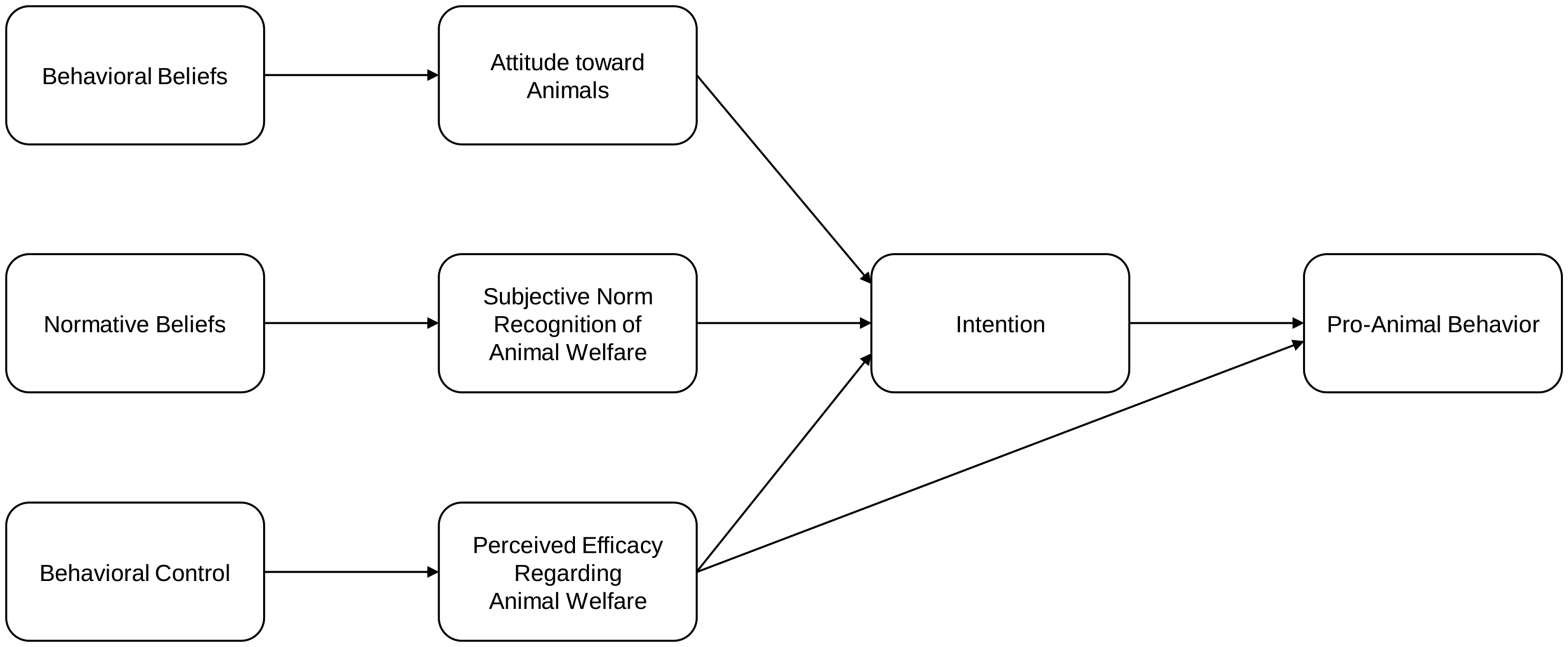
Hypothetical framework of the present study.

The hypothetical framework of this study is illustrated in Figure 2.

## 3 Methods

### 3.1 Data collection and samples

The data for empirically testing the hypotheses were obtained through a nationwide online sample survey targeting South Korean adults aged 19 or older using an online panel. After a pretest to check the reliability of the questionnaire, participants were recruited between 20 August and 1 September 2021. Prior to data collection, ethical approval was obtained from the Institutional Review Board (IRB No. 2107/003-010). Informed consent was obtained electronically from all respondents before participation. The survey commenced only after participants indicated their consent by checking (clicking) the agreement option. A sample of 2,000 responses in total was collected using the quota sampling method, ensuring good representation of the national population across key demographic variables including age, gender, and region. Nevertheless, as with other internet-based surveys, there remains a possibility of bias arising from the underrepresentation of populations lacking internet access or from social desirability effects. To assess general perceptions of animal welfare and related policies, participants were asked to respond to a series of survey items. Specifically, three statements measured their perceptions of: (1) the primary value of animal welfare policies, (2) the perceived current level of animal welfare under Korean policy frameworks, and (3) the anticipated future importance of national policies for animal welfare. Responses were recorded either by selecting from a list of optional statements or by rating agreement on a 5-point Likert scale ranging from 1 (strongly negative) to 5 (strongly positive).

### 3.2 Model variables and measures

The questionnaires were designed to investigate intentions and behaviors induced from their attitude toward animals (behavioral belief), subjective norm regarding animal welfare (normative belief), and perceived efficacy regarding animal welfare (behavioral control) to establish our model.

#### 3.2.1 Dependent variable

To check the respondents’ engagement in pro-animal behavior, the dependent variable, a questionnaire consisting of 12 items asked if the respondents have participated the following behaviors for the purpose of enhancing animal welfare. The questionnaires were designed to cover various levels of pro-animal behaviors, ranging from very personal actions to public engagement that affects others. The items were: (1) writing or sharing social media posts related to animal welfare, (2) refraining from purchasing meat or dairy products, (3) purchasing animal welfare-certified products, (4) purchasing animal test-free products, (5) refraining from purchasing animal abuse products (e.g., fur or down), (6) supporting animal protection organizations or groups, (7) signing petitions for animal related issues, (8) voting for political candidates suggesting animal welfare policies, (9) participating in protests for animal related issues, (10) being a vegetarian for ethical reasons, (11) adopting an animal from a shelter, and (12) explaining or persuading others about animal welfare actions or policies. The items utilized a dichotomous response format, with respondents selecting “yes” if they had engaged in the specified behavior and “no” if they had not.

#### 3.2.2 Independent variables

##### 3.2.2.1 Attitude toward animals

Respondents’ behavioral belief, reflected in their attitudes toward animals, was measured using the shortened Animal Attitude Scale (AAS-10). AAS-10 is one of the most widely used tools to investigate the ethical and behavioral aspects of human–animal interactions (55). The scale includes 10 statements rated on a 5-point Likert scale ranging from 1 (definitely disagree) to 5 (definitely agree). The total AAS-10 score indicates the respondents’ pro-animal attitude: the higher the score, the stronger the respondent’s ethical stance concerning the use of animals.

##### 3.2.2.2 Subjective norm regarding animal welfare

Respondents’ normative belief was measured by their subjective norm regarding animal welfare in society. Respondents were asked whether they are aware of the shared norms in South Korean society. The items comprised four questions assessing the respondents’ awareness of the following: (1) that animals are no longer legally considered as “objects” under the Korean Civil Code through recent amendments; (2) that South Korea has enacted an Animal Protection Act; (3) that the South Korean government has developed and is implementing a mid- to long-term strategic plan for animal welfare; and (4) that a governmental division dedicated to animal welfare has been established. These items also utilized a dichotomous response format, with respondents selecting “yes” if they were aware of each item and “no” if they do not.

##### 3.2.2.3 Perceived internal and external efficacy regarding animal welfare

Previous research on TPB adopted self-efficacy scales as a proxy measurement for PBC from the very early stages of the theory’s development (44, 49). Self-efficacy is defined as the belief in one’s capabilities to organize and execute the courses of action required to manage prospective situations (39, 56). Using a 4-point Likert scale (1 = strongly disagree to 4 = strongly agree), we measured the respondents’ degree of agreement with items designed to assess their perceived efficacy related to animal welfare (e.g., “I am capable of choosing and purchasing animal welfare-certified livestock products,” “I believe that my choice to purchase animal welfare-certified products will effectively contribute to improving animal welfare.”). The responses were subdivided into internal and external efficacy, with composite scores calculated for subsequent analyses.

#### 3.2.3 Mediating variable

Finally, respondents answered five questions about their behavioral intentions to support animal welfare in the future. These items asked the respondents’ intentions to consider animal welfare when they visit places such as (1) a zoo or aquarium, (2) a festival using animals, (3) an animal café or a petting zoo, (4) a fishing site, and (5) an animal show, including circus or racing. Each item was rated on a 4-point Likert scale from “not important at all” to “very important.” The sum of the scores was calculated to represent the degree of behavioral intentions to support animal welfare.

## 4 Results

### 4.1 Sample characteristics and basic statistics

Table 1 presents the characteristics of the survey sample. The sample consisted of 49.5% (*n* = 990) males and 50.5% (*n* = 1,010) females. The majority of respondents were aged over 60 years, representing 31.1% (*n* = 621) of the total. This distribution closely mirrors the demographic composition of the South Korean population, in which 48.7% are male and 51.3% are female, and the age distribution is 14.6% under 30 years, 15.7% aged 30–39, 18.9% aged 40–49, 18.9% aged 50–59, and 31.9% aged 60 years or older. About 46.6% of the respondent had owned a pet in the last 10 years, potentially influencing their attitudes toward animals. Group differences in pro-animal attitudes were observed, with the lowest levels found among males, older adults (60 and above), and individuals identifying as politically conservative.

**Table 1:** Sample characteristics.

Individual characteristics		N (%)	Pro-animal attitude M (SD)	Sig.
Gender	Males	990 (49.5)	2.11 (5.20)	t = −13.720 (*p* < 0.001, Cohen’s d = 5.19)
	Females	1,010 (50.5)	5.30 (5.18)	
Age	Under 30	279 (14.0)	4.24 (5.61)^a^	*F* = 7.248 (*p* < 0.001, η^2^ = 0.014)
	30–39	316 (15.8)	4.45 (5.88)^a^	
	40–49	392 (19.6)	4.18 (5.11)^a^	
	50–59	392 (19.6)	3.74 (5.16)^a^	
	60 and above	621 (31.1)	2.81 (5.36)^b^	
Political orientation	Conservative	421 (21.1)	2.61 (5.94)^a^	*F* = 11.493 (*p* < 0.001, η^2^ = 0.011)
	Moderate	1,017 (50.8)	3.93 (5.10)^b^	
	Progressive	562 (28.1)	4.16 (5.50)^b^	
Residential area	Rural	163 (8.2)	3.82 (5.56)	*F* = 0.301 (*p* = 0.740)
	Suburban	961 (48.0)	3.64 (5.31)	
	Urban	876 (43.8)	3.59 (5.40)	
Pet ownership (within 10 years)	No	1,069 (53.4)	2.91 (5.37)	t = 7.183 (*p* < 0.001, Cohen’s d = 5.365)
	Yes	931 (46.6)	4.64 (5.35)	
Household income (monthly)	Under $2,000	212 (10.6)	3.95 (5.35)	*F* = 1.790 (*p* = 0.112)
	$2,000–4,000	609 (30.4)	3.40 (5.42)	
	$4,000–6,000	539 (27.0)	3.64 (5.36)	
	$6,000–8,000	347 (17.4)	4.33 (5.39)	
	$8,000–10,000	175 (8.8)	3.28 (5.52)	
	over $10,000	118 (5.9)	4.15 (5.79)	
Education	Elementary	19 (1.0)	4.00 (4.76)	*F* = 0.828 (*p* = 0.507)
	Middle	35 (1.8)	4.60 (6.17)	
	High	432 (21.6)	3.93 (5.33)	
	University (cur)	368 (18.4)	3.35 (5.47)	
	University (grad)	1,146 (57.3)	3.72 (5.44)	
Total		2,000 (100.0)	3.72 (5.43)	

Means sharing the same superscript are not significantly different at *p* < 0.05 based on Tukey’s B post hoc comparison. M = mean, SD = standard deviation.

Regarding general perceptions of animal welfare and policies, the largest proportion of participants (36.3%) indicated that animal welfare policies are important because animals are moral beings with intrinsic value. This was followed by the view that animal and human welfare are closely interconnected (28.8%), and the belief that welfare should be improved to reduce suffering in sentient beings (21.2%). Human-centric values—such as the idea that animal suffering causes discomfort to humans (7.3%) or that improved welfare enhances the market value of animal products (6.5%)—were less frequently endorsed as the primary basis for animal welfare policy (see Table 2). In terms of the perceived current level of animal welfare under Korean animal welfare policies, 43.5% of respondents (*n* = 871) believed that national policies do not adequately ensure animal welfare, while only 15.4% responded that welfare is well ensured. Meanwhile, 41.1% considered the level to be comparable to that of other developed countries. As for perceptions of the future importance of national animal welfare policies, a strong majority (82.7%) anticipated that these policies will become more important (18.0% “definitely more,” 64.7% “more than now”). Another 15.8% believed the importance would remain the same, while only 1.5% expressed negative expectations.

**Table 2:** Perceived value orientations associated with animal welfare policies.

Statements	% (n)
Improved animal welfare enhances the market value of animal products.	6.5 (129)
Because animals can feel pain, we should improve their welfare to reduce their suffering.	21.2 (423)
Seeing animals in pain causes discomfort to humans, so we should improve animal welfare.	7.3 (146)
Animal and human welfare are closely interconnected, so a decline in animal welfare can negatively affect human welfare.	28.8 (577)
Animals are moral beings with intrinsic value in their lives, so promoting their welfare is important.	36.3 (725)
Total	100 (2,000)

Survey item: “Which of the following statements best reflects the value you personally associate with animal welfare policies?”

### 4.2 Variables and structural equation model (SEM) analysis

The causal relationships hypothesized in this study (see Figure 2) were analyzed using SEM with STATA 18.5 MP. SEM is particularly suitable for this analysis as it allows for the simultaneous estimation of multiple causal paths and effectively distinguishes among the direct, indirect, and total effects of explanatory variables on dependent variables. SEM has been widely adopted in previous research testing TPB-based models (57).

Since our model was explicitly derived from TPB, we did not conduct an Exploratory Factor Analysis (EFA). Instead, we directly applied SEM. In our analysis, we assumed the causal relationships among latent (i.e., unobserved) variables as predicted by the TPB (see Supplementary Table 1 for the Confirmatory Factor Analysis for each item).

Table 3 summarizes the basic descriptive statistics for the variables incorporated in the model. As previously explained, the variables were measured using multiple items, and most of the scales demonstrated good reliability, with the exception of the items for measuring subjective norm, which exhibited a marginal level of reliability.

**Table 3:** Descriptive statistics for the variables in the structural equation model.

Variable		# of observations	Mean (SD)	Cronbach’s α	# of items
Dependent	Behavior	2,000	15.17 (2.76)	0.800	12
Mediate	Intention	2,000	14.04 (3.21)	0.865	5
Independent	Attitude (AAS)	2,000	33.72 (5.43)	0.792	10
	Subjective norm	2,000	5.96 (1.18)	0.592^*^	4
	Internal efficacy	2,000	19.11 (3.51)	0.819	7
	External efficacy	2,000	13.22 (2.35)	0.735	5

^*^The low reliability may stem from dichotomous response and the varying levels of knowledge required by the items. Assessing the reliability of the items with McDonald’s ω, which relaxes the assumption of equal factor loadings, yielded an improved estimate than Cronbach’s α (0.600).

Overall, the goodness-of-fit indices suggest a satisfactory model fit, as summarized in Table 4. The absolute fit indices indicate good model fit (RMSEA = 0.048, SRMR = 0.053), while relative fit indices show a moderate level of model fit (CFI = 0.837, TLI = 0.827), falling below the conventionally recommended threshold of 0.90 for good fit (58). Although the CFI and TLI values did not meet the conventional criterion, the RMSEA and SRMR indicate a good model fit. RMSEA reflects the degree to which the model estimates the population covariance structure, and SRMR captures the average discrepancy between observed and predicted correlations. The relatively low CFI and TLI may be attributed to their sensitivity to model complexity which is driven by TPB. However, RMSEA and SRMR provide more favorable evidence of the model’s overall fitness. Taken together, the results suggest that the hypothesized model adequately represents the data structure.

**Table 4:** Goodness of fit indices for the structural equation model.

Fit index	Value	Recommended Threshold
RMSEA	0.048	< 0.06
SRMR	0.053	< 0.08
CFI	0.837	≥ 0.90
TLI	0.827	≥ 0.90

**Figure 3:**
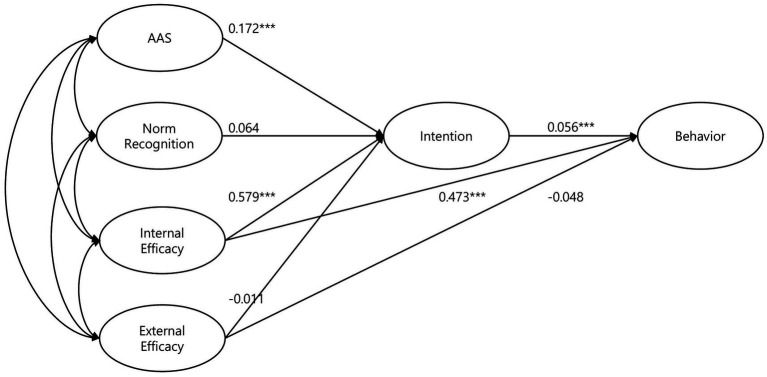
Structural pathways of the structural equation model.

Figure 3 represent the structural path of the model and Table 5 presents the estimated effects of the variables analyzed by SEM. Among the independent variables, attitude measured by AAS-10 had a significant positive effect on the intention to consider animal welfare (β = 0.172, *p* < 0.001), confirming hypothesis H1. However, subjective norm did not have a significant influence on intention (β = 0.064, *p* = 0.470), rejecting hypothesis H2. The results were mixed for behavioral control, which was measured through perceived efficacy consisting of two dimensions, internal efficacy and external efficacy. The results of the SEM analysis show that internal efficacy had a strong effect on intention (β = 0.579, *p* < 0.001), but external efficacy had an insignificant effect (β = −0.011, *p* = 0.868), showing only confirmation of H3-1 but not H3-2.

**Table 5:** Estimated effects of the structural equation model.

Variable	Hypothesis	Unstandardized B	SE	Standardized *β*	*p*-value
Intention					
Attitude (AAS)	H1	0.172	0.035	0.162	< 0.001
Subjective norm	H2	0.064	0.089	0.026	0.470
Internal efficacy	H3-1	0.579	0.094	0.343	< 0.001
External efficacy	H3-2	−0.011	0.064	−0.007	0.868
Behavior					
Intention	H4	0.056	0.011	0.138	< 0.001
Internal efficacy	H5-1	0.473	0.036	0.691	< 0.001
External efficacy	H5-2	−0.048	0.025	−0.080	0.055

With respect to the dependent variable, the intention to consider animal welfare was significantly associated with pro-animal behavior, accepting hypothesis H4. People who care more about animal welfare in their everyday lives were more likely to engage actual pro-animal behavior (*β* = 0.056, *p* < 0.001). It is also noteworthy that internal efficacy had a positive effect on behavioral outcome not only through the indirect path via intention but also through a direct path (β = 0.473, p < 0.001), confirming hypothesis H5-1. In contrast, the direct effect of external efficacy on behavioral outcome was found to be statistically insignificant (β = −0.048, *p* < 0.055), rejecting hypothesis H5-2.

## 5 Discussion

### 5.1 Main findings and implications

Based on participants’ responses, public perceptions of animal welfare and related policies indicate that the primary value of such policies lies more in protecting animals’ intrinsic value and sentient capacities than in promoting human-centered benefits. Moreover, further improvements in animal welfare standards are anticipated, along with higher recognition of the importance of related national policies. These findings underscore an increasing public demand for appropriate and effective political engagement in animal welfare issues, consistent with findings from previous studies (4, 27, 36, 59).

This research employed the TPB framework to investigate the role of behavioral beliefs, normative beliefs, and behavioral control in shaping pro-animal behaviors. Structural equation modeling revealed that attitude toward animals and internal efficacy were the strongest predictors of intention and behavior. By contrast, subjective norm and external efficacy were not significant, with external efficacy even showing a slightly negative but non-significant association. These findings highlight that pro-animal behavior in Korea is primarily driven by individual motivation and perceived internal efficacy rather than external pressures or institutional guidance.

The results underscore the importance of internal motivation in shaping pro-animal behavior (60–62). Intention appears to be formed more through personal pro-animal attitude and self-confidence than through normative pressure. The lack of significance for subjective norm may reflect the limited salience of social or institutional expectations in this domain, or due to the narrowing operationalizing into perceptions of official norms.

The findings on the effect of perceived efficacy were mixed; internal efficacy had a strong effect on pro-animal behavior both directly and indirectly, whereas external efficacy had no significant effect on intention and behavioral outcome. Although only slightly not significant, it is noteworthy that the estimated effect of external efficacy on pro-animal behavior was negative, contrary to the prediction in H5-2 and somewhat counter intuitive. This result implies a potential mediation effect by internal efficacy between external efficacy and behavioral outcomes (43). External efficacy can play a role as a double-edged sword. On one hand, higher external efficacy with institutional trust may directly lead displacement effect to decrease behavior with lower individual responsibility. On other hand, a stronger belief system responsiveness to animal welfare may indirectly promote pro-animal behaviors by enhancing perception on individual’s internal confidence and capability. Therefore, to translate intention into actual behavior, not only institutional official support but also micro level internal motivating process is needed. This results align reasonably well with the study of Vermeir and Verbeke (31) that highlights publics behavioral outcome would be differentiated by how institutional or political infrastructure contribute individual motivation.

Practically, these findings have important implications for developing strategies aimed at enhancing people’s pro-animal behavior. The findings suggest that voluntaristic motivation and a sense of agency play a central role in encouraging pro-animal behavior. This does not imply, however, that legislative efforts are ineffective in promoting pro-animal behavior. Rather, they highlight that individual motivation and normative standards can fortify each other. Robust social norms, either formally codified as laws or informally established as cultural expectations, can ensure that individuals in society maintain at least minimum standards for animal welfare consideration. Systematic approaches to enhance people’s intention to consider animal welfare and engagement in pro-animal behaviors should be coupled with education and the cultivation of voluntary awareness regarding responsibility toward animals (63, 64). In the Korean context animal welfare policy and institutions —such as the Animal Protection Act, welfare certification schemes, and the Comprehensive Animal Welfare Plan—have often developed in fragmented or externally imitative ways, which may have weakened public trust and lowered perceptions of external efficacy. Strengthening policy responsiveness and transparency could thus complement individual motivation by fostering broader public confidence.

### 5.2 Limitations

This study also has several limitations, particularly concerning measurements, that should be addressed in subsequent research. First, pro-animal behaviors were assessed with the 12-items encompassing different level of psychological engagement or cost, which may over simplify the construct of engagement in pro-animal behavior. Future research should more clearly distinguish levels of engagement in behavioral measurement.

Second, we also acknowledge the possibility that the way perceived subjective norms were assessed may have partially contributed to the non-significance of the items in the SEM model. Since we focused primarily on perceptions of official norms, pressure from the media, peer group, or other significant others, which appeared effective in the previous research on TPB, were not captured. In addition, use of 4 dichotomous response categories may have reduced the reliability of the items. Future research should employ more nuanced measures to improve both the validity and reliability of the scale, and to enhance the overall model fit.

As seen in Table 1, pro-animal attitudes differ by age group. This pattern indicates that the structural relations captured by our models may vary across age groups, and suggests that alternative specifications may better account for responses among older generations. That is, distinct motivators could underlie pro-animal behaviors in those age group. Accordingly, we caution against overgeneralization and recommend future research examine age-specific mechanisms through stratified or multigroup analyses.

### 5.3 Further research

Future studies should refine the measurement of pro-animal behavior and subjective norm, and explore generational differences through stratified analyses. To expand on the findings of the present study, research should examine how individual-level dispositions interact with macro-level policies. A substantial amount of prior research has demonstrated how policies interplay with personal disposition in the realm of pro-animal behavior (10, 20, 59, 65). In this regard, policy feedback theory (PFT) provides a useful theoretical framework for exploring the macro-level impact of existing policies and institutional frameworks on public attitudes, behavior, and support for future policy changes (66–68). Integrating TPB and PFT could yield a more comprehensive account of how internal motivation and institutional arrangements foster sustainable pro-animal engagement.

## 6 Conclusion

This study advances research on public awareness and behavior regarding animal welfare by providing insight on not only perception of animal welfare and related policies but also the individual-level predictors of pro-animal behavior through the socio-psychological lens of the TPB framework. Though the model mainly focuses on individual’s psychological mechanism, the logical path to the dependent variable elucidates how pro-animal behaviors can be elicited from psychological factors.

The results underlined the considerably rising public demand for appropriate and effective political engagement in animal welfare issues. Our analysis revealed the critical role of individual beliefs in shaping intentions and behavioral outcomes related to animal welfare, underscoring the importance of considering individual-level factors in shaping public attitude toward animal. Notably, internal motivation and perceived control—specifically, attitudes toward animals and internal efficacy—emerged as primary drivers of the intention to consider animal welfare, rather than subjective norm and external efficacy. Though we do not directly test the role of policies here, this finding indirectly suggest that why pro-animal policies should complement the development of institutional frameworks, such as laws and regulations, with targeted interventions geared at fostering people’s intrinsic care and responsibility towards animals to enhance public awareness and promote pro-animal behaviors.

In sum, this research highlights the value of individual-level approaches in animal welfare research and policy design, while suggesting the integration of policy feedback theory as a direction for further explorations on the dynamic interplay between institutional frameworks and personal disposition.

## Data Availability

The raw data supporting the conclusions of this article will be made available by the authors, without undue reservation.
