# Correction: Understanding public engagement in animal welfare in South Korea: a theory of planned behavior approach

**DOI:** 10.3389/fvets.2026.1766402

**Published:** 2026-01-30

**Authors:** Seola Joo, Myung-Sun Chun, Hyomin Park

**Affiliations:** 1 Research Institute for Veterinary Science, College of Veterinary Medicine, Seoul National University, Seoul, Republic of Korea; 2 Department of Urban Sociology, College of Urban Science, University of Seoul, Seoul, Republic of Korea

**Keywords:** animal welfare, policy, theory of planned behavior, animal attitude scale, structural equation modeling, self-efficacy

Supplemental material Table 1 was omitted. The file(s) have now been published.

The original version of this article has been updated.

