# Supplementary material for: Understanding public engagement in animal welfare in South Korea: a theory of planned behavior approach
Source: Front Vet Sci. 2025 Nov 17;12:1657203. doi: 10.3389/fvets.2025.1657203 (PMC12668202; doi:10.3389/fvets.2025.1657203)
Supplement: Supplementary file 1 [file Table_1.docx]

Supplementary Table 1. Result of Confirmatory Factor Analysis for Each Measurements

| **Fit Index** | **Attitude(AAS)** | **Subjective Norm** | **Internal Efficacy** | **External Efficacy** | **Intention** | **Behavior** |
| --- | --- | --- | --- | --- | --- | --- |
| RMSEA | 0.122 | 0.053 | 0.103 | 0.071 | 0.126 | 0.083 |
| SRMR | 0.078 | 0.021 | 0.049 | 0.028 | 0.033 | 0.059 |
| CFI | 0.771 | 0.986 | 0.926 | 0.973 | 0.965 | 0.842 |
| TLI | 0.705 | 0.958 | 0.889 | 0.945 | 0.930 | 0.807 |
